# Design and Validation of a Method to Determine the Position of the Fovea by Using the Nerve-Head to Fovea Distance of the Fellow Eye

**DOI:** 10.1371/journal.pone.0062518

**Published:** 2013-05-07

**Authors:** Mathijs A. J. van de Put, Fara Nayebi, Danna Croonen, Ilja M. Nolte, Wouter J. Japing, Johanna M. M. Hooymans, Leonoor I. Los

**Affiliations:** 1 Department of Ophthalmology, University Medical Center Groningen, University of Groningen, Groningen, The Netherlands; 2 Department of Epidemiology, University Medical Center Groningen, University of Groningen, Groningen, The Netherlands; University of Tennessee, United States of America

## Abstract

**Purpose:**

To measure the nerve-head to fovea distance (NFD) on fundus photographs in fellow eyes, and to compare the NFD between fellow eyes.

**Methods:**

Diabetic patients without retinopathy, (n = 183) who were screened by fundus photography at the University Medical Center Groningen, the Netherlands from January 1^st^ 2005 until January 1^st^ 2006 were included. The NFD was measured in left and right eyes both from the center and from the rim of the nerve-head. To determine inter- and intra-observer agreement, repeated measurements by one observer (n = 3) were performed on all photographs and by two observers on 60 photographs (30 paired eyes). The effect of age, gender, and refractive error on NFD was analysed.

**Results:**

The correlation of NFDs between the left and the right eye was 0.958 when measured from the center of the nerve head (mean difference 0.0078 mm. ±SD 0.079 (95% limits of agreement −0.147–0.163)) and 0.963 when measured from the rim (mean difference 0.0056±SD 0.073 (95% limits of agreement −0.137–0.149)). Using the NFD between fellow eyes interchangeably, resulted in a standard error of 0.153 mm. Intra- and inter-observer variability was small. We found a significant effect of age (center of the nerve-head (P = 0.006) and rim of the nerve head (P = 0.003)) and refractive error (center of nerve-head (P<0.001) and rim of nerve head (P<0.001)) on NFD.

**Conclusions:**

The NFD in one eye provides a confident, reproducible, and valid method to address the position of the fovea in the fellow eye. We recommend using the NFD measured from the center of the nerve-head since the standard error by this method was smallest. Age and refractive error have an effect on NFD.

## Introduction

In macula-off rhegmatogenous retinal detachment (RRD), visual recovery is highly variable, even after successful reattachment of the macula[1]–[3]. The height of macular detachment has been coined as a potential factor influencing visual recovery[4]–[5]. Height of macular detachment is defined by the distance between the fovea and the retinal pigment epithelium and can be measured by ultrasonography[4]–[5].

Because of its resolution, it is impossible to recognise the foveal dip by ultrasonography[6]–[8]. The nerve-head can be recognised by ultrasonography, and may thus serve as a landmark for foveal position, provided the nerve-head to fovea distance (NFD) is known[6]–[12]. Physiologically, the NFD varies between individuals[9]–[12]. Factors known to influence the NFD include developmental disturbances, [13] foci of chorioretinitis, [13] fibrous traction bands, [13] an unequal distribution of retinal vessels,[14]–[15] an uneven distribution of collagen tissue in the lamina cribrosa, [16] and a tilted or rotated nerve-head[17]–[23]. Also, age, gender and refraction possibly influence the NFD [11]. Since it is impossible to make direct measurements of the fundus of a living eye, information on an individual NFD must be obtained by measurements of an image of the fundus [24]. This can be difficult when changes in the position of the fovea as in macula-off RRD interfere with an imaging technique[3]–[5]. While there is considerable variation in NFD between individuals, both NFDs within one individual are correlated[10]–[12] We evaluated whether the NFDs measured on a fundus photograph of one individual could be used interchangeably between both eyes to obtain a valid method to determine the position of the fovea in macula-off RRD by ultrasonography.

Such a method enables our research group to precisely determine the distance between the fovea and the retinal pigment epithelium in macula-off RRD in our research project on the possible relationship between recovery of visual function and height of macular detachment. This method could also be adapted for use in optical coherence tomography based studies on foveal thickness in situations of unilateral pathology where the fovea cannot be recognised morphologically because of diffuse thickening of the macula and central fixation may be affected by the macular pathology. Examples hereof include subretinal neovascularisation, diffuse diabetic macular edema, and diffuse macular thickening associated with an epiretinal membrane. A prerequisite in these situations would be the relative normality of the fellow fovea. In addition, it would be interesting to evaluate whether anatomical symmetry with regard to NFDs exists between fellow eyes.

## Methods

### Study Population

Retrospectively, we selected 400 diabetic patients who were enrolled in our diabetic screening program and underwent routine examination involving a fundus photograph of both eyes once yearly at the University Medical Center Groningen from January 1^st^, 2005 until January 1^st^, 2006 from our IMAGEnet 2000™ 2.53© database (Topcon™ Europe BV, Leicestershire, UK) for Windows 2000™ digital imaging system (Microsoft™ Corp, SF, Cal, US). The patients were chosen in such a way that the number of patients were approximately equal in seven age groups (20–29, 30–39, 40–49, 50–59, 60–69, 70–79, and 80–89 years of age). The research adhered to the tenets of the Declaration of Helsinki and the Ethics Committee of the University Medical Center Groningen decided that approval was not required for this study. All 400 patients were asked to sign an informed consent form. Patients were excluded when written consent was not obtained (n = 174) or when the quality or field of view of one of the fundus photographs prevented accurate measurements (n = 11). In addition, all patients with diabetic retinopathy, ophthalmologic congenital malformations, retinal or choroidal scars, or a more than 45° rotated nerve-head on photographic imaging were excluded (n = 16)[13]–[23]. Therefore, our study population consisted of 199 patients. Information on age, gender, visual acuity (VA), refraction, and prior cataract extraction (CE) was obtained from the patients’ charts. Patients with an uncorrected Snellen VA of ≥0.8 were assumed to be emmetropic.

### Measurements of Nerve-head to Fovea Distance

Digital fundus photographs were made by two experienced medical photographers, 30 minutes after the administration of one drop of tropicamide 0.5% and one drop of phenylefrine 2.5% in both eyes, using a xenon lamp for illumination of 300 WS at the maximum, under a 50° angle, using the TRC-50 IX fundus camera, (Topcon™ Europe B.V., Leicestershire, UK).

**Figure 1:**
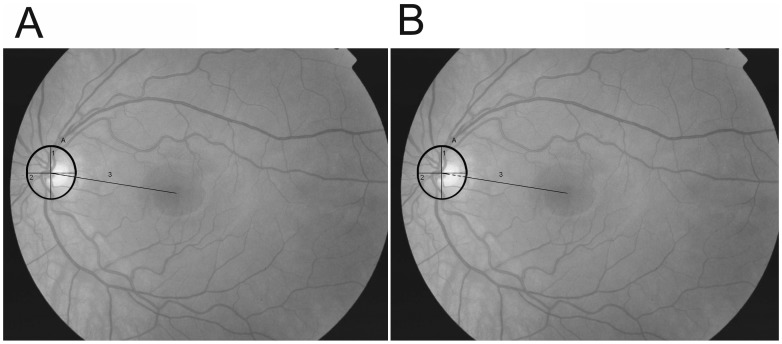
A. Nerve-head to fovea distance measured from the center (A) and B. from the rim (B) of the nerve-head.

On each fundus photograph, the circumference of the optic nerve-head was manually marked using the software program IMAGEnet™ 2000 2.53©. The observers were instructed to take the edge of the optic nerve head and not the peripapillary atrophy region (if present). Major and minor axes were drawn manually on the marked circumference of the optic nerve head. The axes were defined as the longest vertical and horizontal diameters. The position of the fovea was visually identified as the darkest appearing spot at the center of the macular area. Then, two lines were drawn manually; one from the intersection of the major and minor axis and one from the border of the optic nerve head (Fig. 1).

Observer 1 (FN) made three repeated measurements of both NFD lengths in both eyes of all subjects in succession to mirror the clinical approach to multiple measurements taken serially. This method decreases the chance of outliers, as divergent measurements are more easily identified. For analysis of agreement of NFD between fellow eyes the average of the three repeated measurements was taken. A standard error, defined as the difference between the 95% limits of agreement and the mean difference, ≤0.2 mm was considered clinically sufficient to implement this method as this is the lateral resolution of our ultrasonography instrument (ultrasonography B 5.0 Quantel medical, France). To determine interobserver variability regarding manually drawing lines at and making measurements on fundus photographs, observer 2 (LIL) also made three repeated measurements on both NFD lengths in both eyes of thirty subjects enrolled in our study independent of observer 1. In all repeated measurements, the circumference of the optic nerve head, major and minor axes, and the two lines between nerve head and fovea were drawn again, and the measurements were fully repeated.

### Magnification

Uncorrected length measurements on disc photos are unreliable because of variations in the degree of magnification[24]–[28]. Magnification strongly depends on the vergence of the internal axis of the eye [28]. The true image size *T* can be calculated by multiplying, the image size *I* at the photograph with, the camera constant *k*, and the refractive power of the human eye *D*: [27], [29]

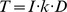



Our camera system uses this formula to calculate the true image size. However, the system assumes that the eye is emmetropic, *i.e.* it assumes an eye refractive power of *D* = 60 diopter (dpt). For an ametropic eye one has to correct the magnification factor of the eye/camera system. In these cases the true image size 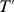
can be calculated by multiplying the true image size *T* determined by the camera software with a corrective factor given by: [27], [29] 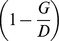, where *G* is the glass refraction of the ametropic eye:
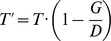



Because the true refraction of patients who had undergone CE was unknown, these patients (n = 16) were excluded from further analysis, resulting in a final study population of 183 subjects.

### Statistical Analysis

Outlier analysis was performed to identify divergent measurements. Mean, standard deviations (SD) and ranges of the NFD were calculated for both eyes. A paired t-test was used to compare refraction differences between eyes. To test for agreement between the NFDs in fellow eyes and between repeated measurements made by different observers on fundus photographs, we made diagnostic plots as proposed by Bland and Altman [29] and calculated the Pearson’s correlation coefficients, mean, SD and the 95% limits of agreement and the 95% confidence interval (CI) for the 95% limits of agreement. Intra- and inter-observer agreement was determined to check the validity of the NFD measurements using Bland and Altman diagnostic plots and the 95% limits of agreement [29]. Differences between intra- and inter-observer measurements were tested using repeated measurements analysis of variance.

A Student’s t-test was performed to compare gender differences in NFD. Linear regression analysis was performed to determine the influence of age and refractive error on NFD. For these analyses the dependent variable was the NFD averaged over the six repeated measurements of both eyes. P-values <0.05 were considered to be statistically significant. Statistical analyses were performed using SPSS software version 16.0© (SPSS inc, Chicago, Ill, US).

## Results

**Figure 2:**
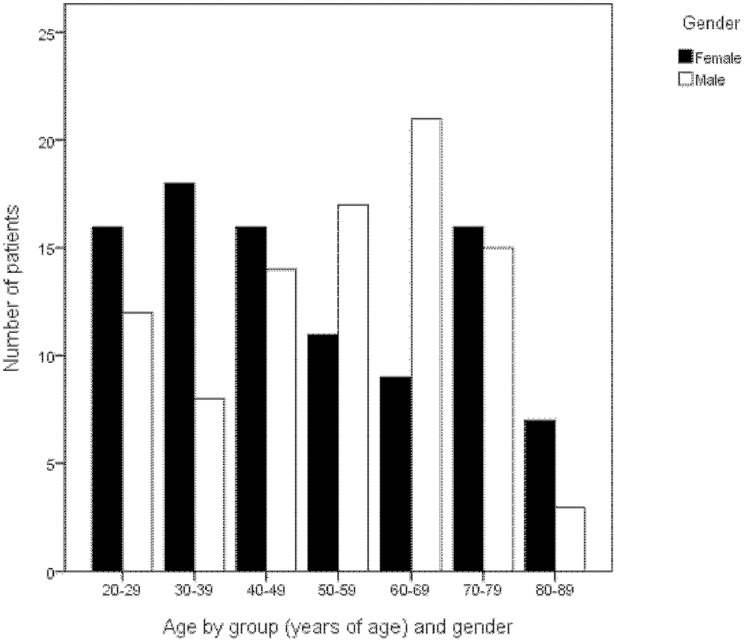
Distribution of age and gender in 183 individuals.

Within our study population (age range 20–87yrs), age groups (20–29, 30–39, 40–49, 50–59, 60–69, and 70–79 years of age) had similar numbers of patients, whereas age group 80–89 had slightly lower numbers than the other groups (Fig. 2). Mean age was 52 years. A similar number of males and females were included (49.2% male: 50.8% female). Table 1 shows the characteristics of the refraction for the 183 pairs of eyes and the characteristics of NFDs measured from the center and the rim of the nerve-head in 183 right and left eyes. There was no significant difference between refractive errors in both eyes. The median difference in refractive error was 1.28 dpt. (range 0.06–7.06 dpt.). Outlier analysis on three repeated measurements for both distances in each eye identified one outlier. We could not find any probable cause for this outlier. Therefore we excluded this measurement from further analysis.

**Table 1:** Characteristics of study population (n = 183); Gender, mean, standard deviations (SD) and range for refractive errors in diopters (dpt) and nerve-head to fovea distance (NFD) measured from the rim and the center of the nerve-head in 183 right eyes (OD) and left eyes (OS) in mm.

Measurement	N	Gender m:f	Mean	SD	Range	≤−5dpt	>−5<0dpt	0dpt	>0<5dpt	≥5dpt
*Refraction OD*	183	90∶93	−0.12	1.6	−7.50–6.50	3	45	90	44	1
*Refraction OS*	183	90∶93	−0.11	1.6	−7.50–6.25	4	43	91	44	1
***NFD Center of the*** ***nerve-head***	**N**	**Gender m:f**	**Mean**	**SD**	**Range**					
*OD*	183	90∶93	4.73	0.28	4.04–5.39					
*OS*	183	90∶93	4.72	0.27	4.00–5.33					
***NFD Rim of the*** ***nerve-head***	**N**	**Gender m:f**	**Mean**	**SD**	**Range**					
*OD*	183	90∶93	3.87	0.27	3.17–4.48					
*OS*	183	90∶93	3.86	0.27	3.08–4.48					

### Nerve-head to Fovea Distance

**Figure 3:**
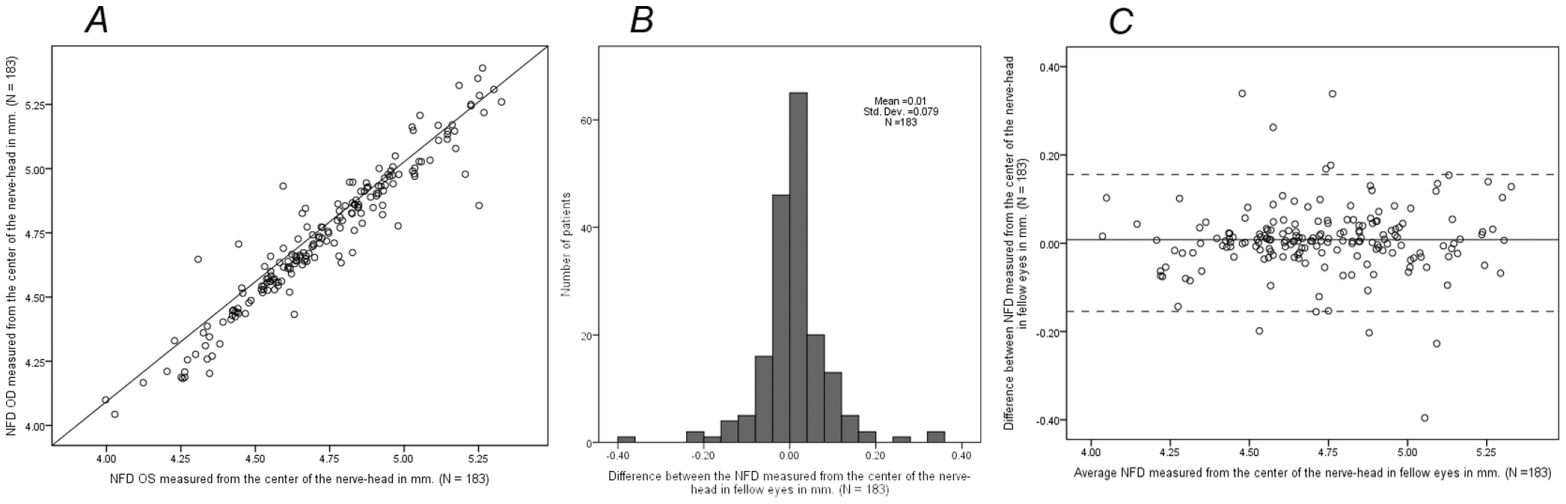
A. NFD measured from the center of the nerve-head of the right eye (OD) plotted against this measurement of the left eye (OS) together with the line of equation (N = 183). **B.** The distribution of differences between the NFDs measured in fellow eyes from the center of the nerve-head (N = 183). **C.** The difference between NFDs against NFD averaged over both eyes measured from the center of the nerve-head between fellow eyes. The solid line indicates the mean and the dotted lines the 95% limits of agreement (N = 183).

Figure 3 shows the diagnostic plots of agreement of NFDs measured in fellow eyes from the center of the nerve-head. Nine measurements (4.9%) made from the center of the nerve-head, and 12 measurements (6.6%) made from the rim of the nerve-head, were outside the 95% limits of agreement and no relationships between the mean and the difference were observed indicating that the measurement errors are normally distributed as required. The correlation of NFDs between fellow eyes was 0.958 when measured from the center of the nerve head and 0.963 when measured from the rim (Table 2). The average differences in NFD and the corresponding 95% limits of agreement in case of three repeated measurements are given in Table 2. These limits fall within the lateral resolution of our ultrasonography-instrument which is 0.2 mm, and hence the measurements of NFD are interchangeable between left and right eyes. When NFD would have been measured only once, the upper limit of the confidence interval for the upper limit of agreement for NFD measured from the center of the nerve head ( = 0.195 mm) is smaller than 0.2, which implies that the error in this measurement is still acceptable.

**Table 2:** Pearson’s correlation coefficient R and agreement measurements for nerve-head fovea distances in left and right eyes.

Measurement	R	Mean	SD^a^	95% Limits of agreement and 95% CI	
*Center of the nerve-head*				*Lower limit & 95% CI*	*Upper limit & 95% CI*
Three repeated measurements	0.958	0.0078	0.079	−0.147 (−0.167, −0.127)	0.163 (0.143, 0.182)
Single measurement			0.085	−0.159 (−0.180, −0.137)	0.174 (0.153,0.195)
***Rim of the nerve-head***				***Lower limit & 95% CI***	***Upper limit & 95% CI***
Three repeated measurements	0.963	0.0056	0.073	−0.137 (−0.156, −0.119)	0.149 (0.130, 0.167)
Single measurement			0.079	−0.150 (−0.170, −0.130)	0.161 (0.141,0.181)

a SD for single measurements = √ [(SD difference for average of three measurements)^2^+ (SD within three measurements of observer 1)^2^+ (SD within three measurements of observer 2)^2^]

**Figure 4:**
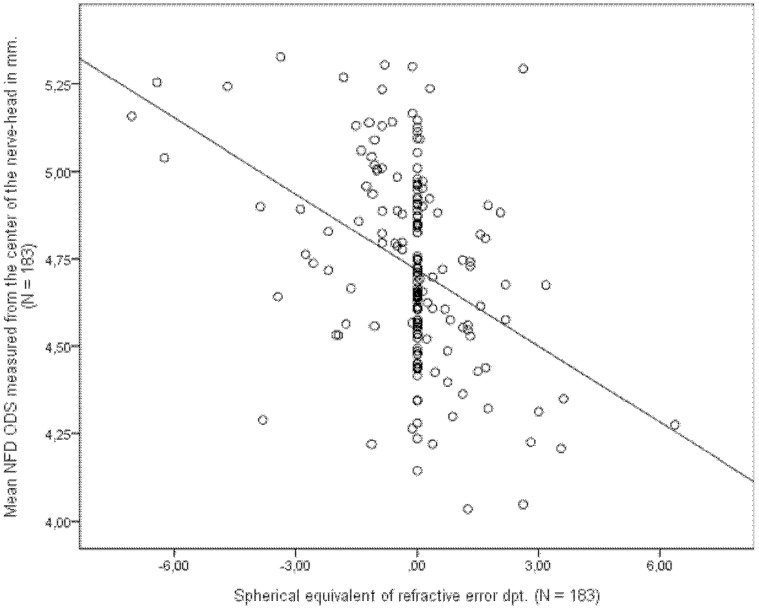
The spherical equivalent of the refractive error of 183 patients plotted against the mean NFD measured form the center of the nerve-head of both eyes (ODS) together with the line of equation (N = 183).

**Figure 5:**
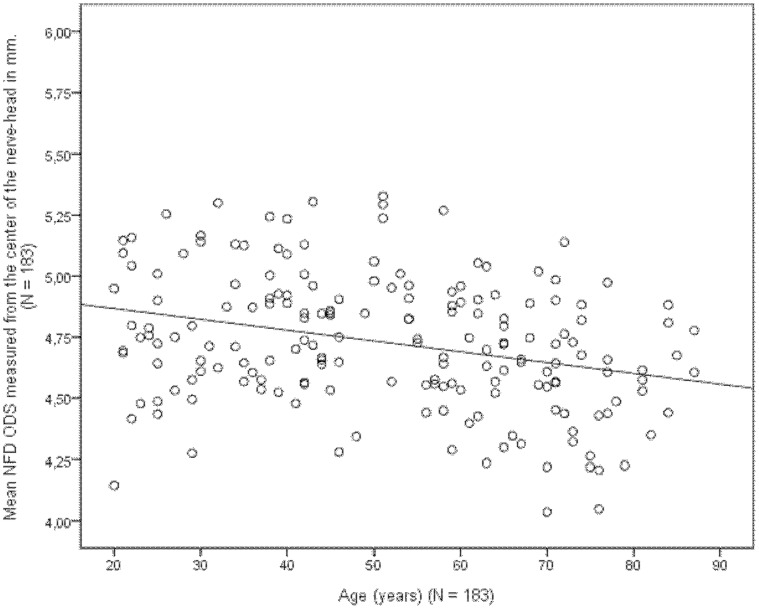
The age of 183 patients plotted against the mean NFD measured form the center of the nerve-head of both eyes (ODS) together with the line of equation (N = 183).

There was no significant difference in NFD between males and females. There was a significant effect of age and refraction on NFD. When measured from the center of the nerve-head, we found that NFD decreased by 0.062 mm (P<0.001) per unit increase in spherical equivalent of refraction (Figure 4), and NFD decreased with aging by 0.0029 mm (P = 0.006) per year of age (R^2^ = 0.206) (Figure 5). When measuring NFD from the rim of the nerve-head we observed a decrease in NFD by 0.050 mm (P<0.001) per unit increase in spherical equivalent of refraction and by 0.0031 mm (P = 0.003) per year of age (R^2^ = 0.165).

### Validity of Measurements

**Figure 6:**
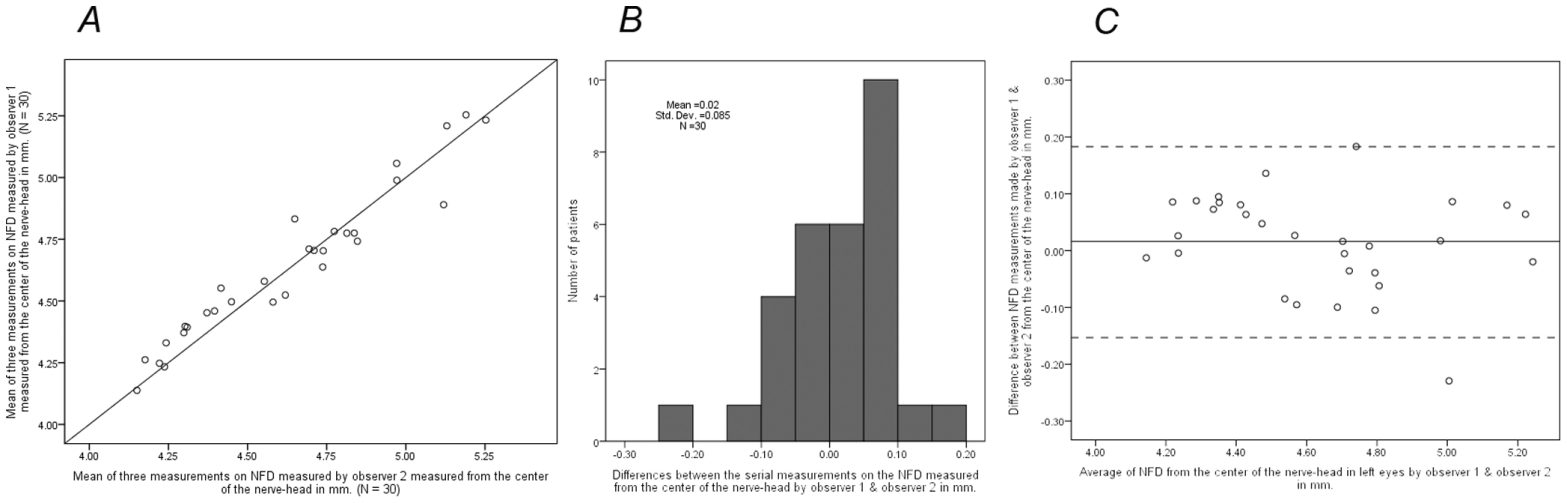
A. NFD made by observer 1 plotted against this measurement made by observer 2 together with the line of equation. **B.** the distribution of differences of the NFDs between the two observers. **C.** The difference between NFDs against NFD averaged over both observers. The solid line indicates the mean and the dotted lines the 95% limits of agreement.

No intra-observer difference between the three measurements was observed for both observers (Table 3). Inter-observer differences were significant for NFD measured from the rim of the nerve-head for both the left eye (P = 0.0072) and the right eye (P = 9.3 10^−7^), but not for NFD measured from the center of the nerve-head. Observer 2 measured the distance from the rim of the nerve-head to the fovea 0.164 mm. shorter in the right eye (95% limits of agreement: −0.141–0.468 for a single measurement; −0.120–0.447 for triple measurements) and 0.076 mm. in the left eye (−0.222–0.374 single; −0.206–0.358; triple) (Table 4; Figure 6). The upper limits of agreement were large for all four measurements ranging from 0.141–0.222, with three of them being larger than the lateral resolution of 0.2 mm implying that these measurement errors are unacceptable.

**Table 3:** Intra- and inter-observer differences between nerve-head to fovea distance (NFD) measurements made from the center and the rim of the nerve-head in 30 right eyes (OD) and 30 left eyes (OS).

Measure	Test	Mean Square	F	P-value
*Center of the nerve-head*				
OD	Within observer 1	0.00247	1.47	0.24
	Within observer 2	0.00210	0.94	0.40
	Between observer 1 and observer 2	0.03784	2.60	0.12
OS	Within observer 1	0.00092	0.59	0.56
	Within observer 2	0.00076	0.45	0.64
	Between observer 1 and observer 2	0.01096	0.99	0.33
*Rim of the nerve-head*				
OD	Within observer 1	0.00163	0.99	0.38
	Within observer 2	0.00007	0.022	0.98
	Between observer 1 and observer 2	1.20324	38.40	9.3 10^−7^
OS	Within observer 1	0.00400	2.88	0.064
	Within observer 2	0.00001	0.004	1.00
	Between observer 1 and observer 2	0.26019	8.35	0.0072

**Table 4:** Pearson’s correlation coefficient *R* and agreement measurements for the averages of three measurements made by two observers on the nerve-head to fovea distance (NFD) mm. from the center of the nerve-head and the rim of the nerve-head in 30 right eyes (OD) and 30 left eyes (OS).

Measurement	R	Mean	SD^a^	95% limits of agreement and 95% CI	
				*Lower limit & 95% CI*	*Upper limit & 95% CI*
*Center nerve-head*					
OD					
Three repeated measurements	0.961	−0.029	0.098	−0.222 (−0.283, −0.161)	0.164 (0.103, 0.225)
Single measurement			0.111	−0.247 (−0.316, −0.178)	0.189 (0.120,0.258)
OS					
Three repeated measurements	0.970	0.016	0.086	−0.153 (−0.206, −0.099)	0.184 (0.131, 0.237)
Single measurement			0.098	−0.176 (−0.236, −0.115)	0.207 (0.146,0.267)
*Rim nerve head*					
OD					
Three repeated measurements	0.923	−0.164	0.145	−0.447 (−0.536, −0.357)	0.120 (0.030, 0.209)
Single measurement			0.156	−0.468 (−0.565, −0.372)	0.141 (0.045,0.238)
OS					
Three repeated measurements	0.914	−0.076	0.144	−0.358 (−0.448, −0.269)	0.206 (0.117, 0.296)
Single measurement			0.152	−0.374 (−0.468, −0.280)	0.222 (0.128,0.316)

a SD for single measurements = √ [(SD difference for average of three measurements)^2^+ (SD within three measurements of observer 1)^2^+ (SD within three measurements of observer 2)^2^].

## Discussion

We have shown that NFDs measured on fundus photographs are highly correlated between eyes and moreover that the limits of agreement fall within the acceptable boundary set by the lateral resolution of the B-mode ultrasonography-instrument. This implies that using NFDs from fellow eyes interchangeably provides an applicable, confident, and reproducible method to determine the position of the fovea by ultrasonography. This method can help overcome the experienced difficulties in cases in which an assessment of macular morphology is needed. In addition, we found a high correlation, an equal distribution of differences and good agreement between repeated measurements on fundus photographs when NFD was measured from the center of the nerve-head by the same and by different observers. When NFD was measured from the rim of the nerve-head, we observed an inter-observer difference. Therefore, the latter method was found to be less reliable.

In contrast, we found a broad range of NFDs in our study population illustrating large inter-individual differences in normal eyes. The described differences could be partly explained by the significant correlation between NFD and age and between NFD and refraction. These results show that the use of the described method is a more accurate method to determine the position of the fovea for ultrasonography measurements compared to the use of any fixed NFD.

The good agreement between the NFDs in fellow eyes found in our study is partly supported by previous studies[10]–[12]. Moreover, in our study individual differences in NFD seem to be smaller than those reported by previous studies[10]–[12]. This may be due to differences in study design relating to the study populations and the study method. Our study population was relatively large and consisted of essentially normal eyes (diabetic patients without signs of retinopathy). Possibly confounding factors included refraction, gender, and age in the subgroup over 70 years of age. Refraction and gender were no selection criteria. Mean refraction turned out to be slightly myopic. We found a high agreement between the refractive errors in fellow eyes, and differences in refractive errors between fellow eyes turned out to be small. This implies that our conclusions cannot be extrapolated to persons with significant anisometropia. In the entire group, similar numbers of males and females were included, but there was a somewhat unequal inclusion of males and females in the different age groups. Study populations in previous studies were smaller or not equally distributed with regard to age[10]–[12]. Furthermore, in previous studies, the prevalence of moderate (−0.5 to −5 D) and high myopia (≥−5) was higher and the agreement between refractive error between fellow eyes was unknown[10]–[11].

In addition, differences between our results and those of others could be explained by the method of correcting for magnification[10]–[12]. We corrected for magnification by using the spherical equivalent of the refraction using a formula previously described by Bengtsson[25], [27]–[28], whereas others corrected for magnification by using keratometric data and the spherical equivalent of the refraction using a formula previously described by Littmann [31]. Bengtsson et al. showed in their comparative study that although correcting for magnification using the axial length is the gold standard, other methods to correct for magnification are almost equally accurate[27]–[28]. Correcting for magnification by means of the spherical equivalent of the refraction is the most comprehensive and easy to practice method to correct for magnification[27]–[28]. If correction for the influence of the glass refraction is considered to be unsatisfactory, correction based on measurements of the axial length seems to be the only alternative[27]–[28]. However if ultrasonography has to make sense, other errors must be rectified as well. Therefore we recommend to correct for magnification by the method described by Bengtsson et al[27]–[28].

Our study found a significant positive correlation between increasing myopia and NFD. Previous studies showed either no correlation with myopia or a significant increase in NFD in highly myopic eyes[11]–[12]. With regard to age, we found significantly shorter NFDs with increasing age. In contrast, previous studies found significantly longer NFDs with increasing age, or an absence of such a correlation[11]–[12]. Possible explanations of a shorter NFD with increasing age include a cohort effect or a real effect due to shrinkage of the eye. Assuming a positive correlation between body height and NFD, NFD would gradually increase in younger persons in parallel with an increasing mean body height as measured over the past decennia in the Netherlands (http://statline.cbs.nl/statWeb/publication/?DM=SLNL&PA=37446&D1=0-21&D2=a&VW=T). Alternatively, a slight shrinkage of the eye during a lifetime could occur due to a general shrinkage of connective tissues in aging persons. These explanations remain speculative since our study and previous ones are cross-sectional and therefore do not give direct information on longitudinal changes. Further, our study shows no significant relationship between gender and NFD in concordance with others[11]–[12].

Our study provides limits of agreement, when using NFDs in fellow eyes interchangeably and standard errors can therefore be easily calculated. In contrast, other studies solely provided *Pearson’s* correlation coefficients[10]–[11]. High correlations found when two methods measure similar quantities inform about the validity of the methods, but they fail to inform about the agreement between methods or whether they can be used interchangeably [30].

### Conclusion

In conclusion, we found that the assessment of the position of the fovea by using the NFDs measured on fundus photographs interchangeably between fellow eyes is highly reliable. Differences between observers were the main source of variability, in particular when the NFD was measured from the rim of the nerve-head. This finding, in conjunction with the known accuracy of ultrasonography, should provide those who need to make an assessment of macular height in macula-off RRD with a helpful, confident, reproducible, and valid method.
